# Real‐world data on the efficacy and safety of immune‐checkpoint inhibitors in elderly patients with non‐small cell lung cancer

**DOI:** 10.1002/cam4.5889

**Published:** 2023-03-31

**Authors:** Daisuke Morinaga, Hajime Asahina, Shotaro Ito, Osamu Honjo, Hisashi Tanaka, Ryoichi Honda, Hiroshi Yokouchi, Keiichi Nakamura, Kei Takamura, Fumihiro Hommura, Yasutaka Kawai, Kenichiro Ito, Noriaki Sukoh, Keiki Yokoo, Ryo Morita, Toshiyuki Harada, Taichi Takashina, Tomohiro Goda, Hirotoshi Dosaka‐Akita, Hiroshi Isobe, the Hokkaido Lung Cancer Clinical Study Group Trial

**Affiliations:** ^1^ Department of Respiratory Medicine, Faculty of Medicine and Graduate School of Medicine Hokkaido University Sapporo Japan; ^2^ Department of Respiratory Medicine Sapporo Minami‐Sanjo Hospital Sapporo Japan; ^3^ Department of Respiratory Medicine Hirosaki University, Graduate School of Medicine Hirosaki Japan; ^4^ Department of Respiratory Medicine Asahi General Hospital Asahi Japan; ^5^ Department of Respiratory Medicine National Hospital Organization Hokkaido Cancer Center Sapporo Japan; ^6^ Department of Respiratory Medicine National Hospital Organization Asahikawa Medical Center Asahikawa Japan; ^7^ Department of Respiratory Medicine Obihiro‐Kosei General Hospital Obihiro Japan; ^8^ Department of Respiratory Medicine Sapporo City General Hospital Sapporo Japan; ^9^ Department of Respiratory Medicine Oji General Hospital Tomakomai Japan; ^10^ Department of Respiratory Medicine KKR Sapporo Medical Center Sapporo Japan; ^11^ Department of Respiratory Medicine National Hospital Organization Hokkaido Medical Center Sapporo Japan; ^12^ Department of Respiratory Medicine Teine Keijinkai Hospital Sapporo Japan; ^13^ Department of Respiratory Medicine Akita Kousei Medical Center Akita Japan; ^14^ Department of Respiratory Medicine JCHO Hokkaido Hospital Sapporo Japan; ^15^ Department of Respiratory Medicine Iwamizawa Municipal General Hospital Iwamizawa Japan; ^16^ Department of Medical Oncology Hokkaido University Graduate School of Medicine Japan; ^17^ Research Division of Cancer Immunotherapy Hokkaido University Hospital Japan

**Keywords:** elderly patient, immune‐checkpoint inhibitor, non‐small cell lung cancer

## Abstract

**Purpose:**

Immune‐checkpoint inhibitors (ICIs) are effective against advanced non‐small cell lung cancer (NSCLC). However, whether the efficacy and safety of ICI treatment in elderly patients are similar to those in younger patients is unclear. This study was designed to address this question.

**Methods:**

We enrolled patients who received ICI monotherapy in Japan between December 2015 and December 2017; those ≥75 years of age comprised the elderly group. We compared the efficacy and safety of ICI monotherapy in elderly patients with those in younger patients and explored prognostic factors in elderly patients.

**Results:**

We enrolled 676 patients; 137 (20.3%) were assigned to the elderly group. The median age of the elderly and younger groups was 78 (range, 75–85) and 66 (range, 34–74) years. The median progression‐free survival (4.8 months vs. 3.3 months, *p* = 0.1589) and median overall survival (12.3 months vs. 13.0 months, *p* = 0.5587) were similar between the elderly and younger groups. Multivariate analysis revealed that a significantly better OS in the elderly group was associated with better responses to first‐ or second‐line ICI treatment (*p* = 0.011) and more immune‐related adverse events (irAEs) (*p* = 0.02). IrAEs that led to ICI discontinuation occurred in 34 of 137 patients (24.8%) in the elderly group, and their survival was significantly higher than that in those who did not have irAEs.

**Conclusion:**

ICI is also effective in elderly NSCLC patients, and treatment discontinuation due to irAEs may be a good prognostic marker.

## Graphical abstract

A real‐world data showed that Immune‐checkpoint inhibitor (ICI) therapy in elderly patients with non‐small cell lung cancer (NSCLC) is as effective and safe as non‐elderly patients.

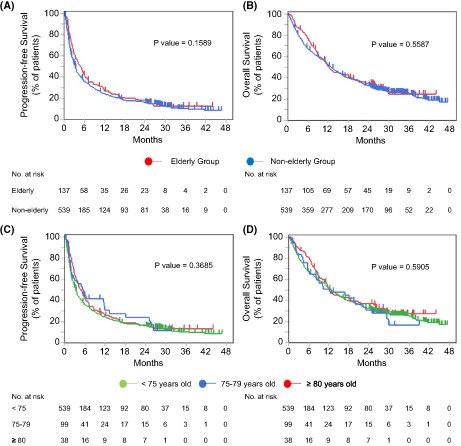

## 1 INTRODUCTION

Lung cancer is the leading cause of cancer‐related death worldwide,^1^ and non‐small cell lung cancer (NSCLC) accounts for approximately 80% of all lung cancers.^2^ In recent years, the number of elderly individuals with cancer has increased owing to population aging, despite advancements in cancer therapy.^3^ The incidence of lung cancer generally increases with age, and approximately half of the patients with lung cancer are over 70 years of age.^4^ Thus, strategies for treating elderly patients with NSCLC are important. The results of several studies have shown that certain cytotoxic agents are safe and effective in elderly NSCLC patients having good performance status (PS).^5^, ^6^, ^7^ Therefore, age alone should not be used to determine whether to administer cytotoxic chemotherapy; instead, the overall systemic condition of the patient, including comorbidities and the Eastern Cooperative Oncology Group (ECOG) PS, should be assessed comprehensively.

Programmed cell death protein‐1 (PD‐1)/programmed death‐ligand‐1 (PD‐L1) inhibitors have been effective in treating advanced NSCLC and thus have become a standard of care.^8^, ^9^, ^10^, ^11^, ^12^ However, despite the increasing incidence and prevalence of cancers among elderly patients, the efficacy and safety of immune‐checkpoint inhibitors (ICIs) have not been fully tested in this specific population. For example, the proportion of patients aged ≥75 years in the CheckMate 017 and 057 studies was <10%, whereas that in the KEYNOTE‐010 study was unknown.^8^, ^9^, ^10^ In a clinical setting, a few studies have examined the efficacy and safety of ICI therapy in elderly patients,^13^, ^14^, ^15^, ^16^, ^17^, ^18^ but most of these studies were single‐arm trials. Thus, it cannot be concluded that ICI therapy is as safe and effective in the elderly as it is in younger patients.

Although the above studies generally show the tendency that ICI therapy is as effective for younger patients, the concept of immunosenescence is still proposed. This logically suggests that ICI may be less effective in elderly patients.^19^, ^20^ This concept is characterized by a dysfunctional immune system represented by T cells.^20^ However, no simple method is currently available to assess immunosenescence in real‐world clinical practice. Therefore, it remains unclear how the immune system of elderly patients can alter their response to immunotherapy.

In a previous study, we retrospectively examined prognostic factors in patients with advanced NSCLC after long‐term anti‐PD‐1 therapy.^21^ Numerous elderly patients were enrolled in that study. Herein, we report a subgroup analysis of the elderly patients to evaluate the efficacy and safety of ICI in elderly NSCLC patients by comparing both parameters with those in younger patients. We also explored potential biomarkers for a good response to ICI in elderly patients.

## 2 MATERIALS AND METHODS

### 2.1 Study design and participants

This is a retrospective, multicenter study of NSCLC patients who started anti‐PD‐1 inhibitor monotherapy between December 2015 and December 2017. We reviewed the medical records of all consecutive patients with advanced or recurrent NSCLC who were treated with nivolumab or pembrolizumab at 15 institutions in the Hokkaido Lung Cancer Clinical Study Group Trial (HOT) in Japan. We did not set any exclusion criteria to avoid selection bias; all patient data were included in the analysis unless essential clinical data were missing. The data cutoff date was December 31, 2019. This study was performed as a subgroup analysis of HOT1902,^21^ was registered at UMIN‐CTR (UMIN000041403), and was approved by the institutional review boards of all involved institutions. The need for informed consent was waived because anonymized data were analyzed. This study was carried out in accordance with The Code of Ethics of the World Medical Association (Declaration of Helsinki). This study was approved by the Hokkaido University Hospital Ethics Committee (approval no. 022–0123).

The cutoff age for defining elderly patients varies; many recent studies use 75 years as the cutoff age.^8^, ^9^, ^10^, ^13^, ^14^, ^15^, ^16^, ^17^, ^18^, ^22^, ^23^ In addition, patients ≥75 years of age are frequently excluded from clinical trials, despite the fact that more real‐world data are needed to study efficacy and safety. Therefore, we defined the elderly group as patients ≥75 years of age, whereas those below 75 years of age were classified as the younger group in this study.

Due to the prolonged time involved in drug approval in Japan, only PD‐1 inhibitors (nivolumab or pembrolizumab) were used as the initial ICI treatment. Data related to the following patient characteristics were collected: age, sex, smoking status, pack‐years, histology, cancer stage, tumor burden, presence of a driver mutation(s), PD‐L1 status, history of radiation therapy within 6 weeks before ICI treatment initiated, steroid administration at the time of ICI treatment initiation, the baseline absolute neutrophil count (ANC), the baseline absolute lymphocyte count (ALC), ECOG PS at the start of the initial ICI treatment, treatment line in which ICI was administered, the clinical response to ICI treatment, the type and grade of immune‐related adverse events (irAEs) that led to ICI treatment discontinuation, and the administration of ICI‐rechallenge treatment. Tumor response was measured using the Response Evaluation Criteria in Solid Tumors (version 1.1).^24^ We also calculated the neutrophil‐to‐lymphocyte ratio (NLR) to identify prognostic factors based on peripheral blood findings.

Because of the study's retrospective design, complete response (CR) and partial response (PR) observations did not require confirmation. Assessments were performed at each participating institution. The tumor burden was defined as the sum of the longest diameters for a maximum of five target lesions and up to two lesions per organ. We only collected information for irAEs that caused treatment discontinuation. For more information on this study, please see our previous article.^21^

### 2.2 Statistical analysis

Categorical data are reported as frequencies (percentages). Continuous data (age, pack‐years, and tumor burden) are reported as medians with ranges. The chi‐square test and Fisher's exact test for independence were used to compare the categorical data. Ages, pack‐years, and tumor burdens were compared using the Wilcoxon rank‐sum test. Progression‐free survival (PFS) was defined as the interval between the initial ICI administration and disease progression or death. Overall survival (OS) was defined as the interval between initial ICI administration and death from any cause. The ICI administration period was calculated from the date of the initial ICI administration to the date of the last administration of ICI therapy. Patients without documented clinical or radiographic disease progression or who were still alive were censored on the date of the last follow‐up. PFS and OS were evaluated using the Kaplan–Meier method and compared using a two‐sided log‐rank test. Hazard ratios (HRs) and 95% confidence intervals (CIs) were estimated using a Cox proportional hazard regression model. The factors considered important based on previous findings and a medical point of view were selected for inclusion in the multivariate analysis, regardless of the results of the univariate analysis. All *p*‐values were two‐sided, and the threshold for statistical significance was set at *p* < 0.05. All statistical analyses were performed using JMP Pro 15 software (SAS Institute Inc).

## 3 RESULTS

### 3.1 Patient characteristics

In this study, we enrolled 676 patients with NSCLC who were administered immunotherapy using PD‐1 inhibitors. The patient characteristics are listed in Table 1. PD‐L1 expression was not investigated in approximately half of the patients. Of the entire group enrolled, 137 patients (20.3%) were in the elderly group, with a median age of 78 years (range, 75–85 years). Most patients in the elderly group were men (*n* = 97, 70.8%), had a smoking history (*n* = 102, 74.4%), had an ECOG PS of 0 or 1 (*n* = 122, 89.1%), and had no known driver mutations (*n* = 119, 86.9%). The median age of the younger group was 66 years (range, 34–74 years). The elderly group included significantly fewer patients with a smoking history (*p* = 0.004) and more patients with a better ECOG PS (*p* = 0.0192). Fewer younger patients than elderly patients received ICI therapy as first‐line therapy (*p* = 0.0216).

**TABLE 1:** Frequencies and types of irAEs in elderly and non‐elderly patients.

	No. (%)			
Characteristic	All	Elderly group	Non‐elderly group	*p‐value* ^a^
	(*n* = 676)	(*n* = 137)	(*n* = 539)	
Age, years				
Median (range)	67 (34–85)	78 (75–85)	66 (34–74)	<0.0001
Sex				0.623
Male	490 (72.5)	97 (70.8)	393 (72.9)	
Female	186 (27.5)	40 (29.2)	146 (27.5)	
Smoking status				0.004
Never smoked	102 (15.1)	35 (25.6)	67 (12.4)	
Current or former smoker	574 (84.9)	102 (74.4)	472 (87.6)	
Pack‐years				
Median (range)	40 (0–330)	30 (0–159)	40 (0–330)	0.0199
≥10	550 (81.4)			
≥30	426 (63.0)	69 (50.4)	357 (63.1)	0.0006
≥50	225 (33.3)			
Histology				0.4703
Adenocarcinoma	415 (61.4)	81 (59.1)	334 (62.0)	
Squamous cell carcinoma	205 (30.3)	47 (34.3)	158 (29.3)	
Non‐small cell lung carcinoma	26 (3.8)	5 (3.7)	21 (3.9)	
Large cell carcinoma	14 (2.1)	3 (2.2)	11 (2.0)	
Others	16 (2.4)	1 (0.7)	15 (2.8)	
Disease stage at diagnosis				0.4834
III	153 (22.6)	36 (26.3)	117 (21.7)	
IV	397 (58.6)	75 (54.7)	322 (59.7)	
Recurrence	126 (18.6)	26 (19.0)	100 (18.6)	
Tumor burden (mm)				
Median (range)	53 (0–330)	49 (0–182)	55 (0–330)	0.2109
Driver mutation				
EGFR mutation	54 (8.0)	16 (11.9)	38 (7.1)	
ALK translocation	5 (0.7)	1 (0.7)	4 (0.7)	
ROS1	5 (0.7)	0	5 (0.9)	
BRAF	3 (0.4)	1 (0.7)	2 (0.4)	
MET	1 (0.1)	0	1 (0.2)	
Others	6 (0.9)	0	6 (1.1)	
Not investigated	602 (89.1)	119 (86.9)	483 (89.6)	
Treatment line				0.0216
1st line	84 (12.4)	27 (19.7)	57 (10.6)	
2nd line	283 (41.8)	54 (39.4)	229 (42.5)	
3rd line and beyond	309 (45.7)	56 (40.9)	253 (46.9)	
ECOG Performance status				0.0192
0	118 (17.4)	26 (19.0)	92 (17.1)	
1	430 (63.6)	96 (70.1)	334 (62.0)	
≥2	128 (18.9)	15 (11.0)	113 (21.0)	
PD‐L1 status (22C3 IHC)				0.3549
<1%	40 (5.9)	7 (5.1)	33 (6.1)	
1%–49%	67 (9.9)	11 (8.0)	56 (10.4)	
≥50%	146 (21.6)	37 (27.0)	109 (20.2)	
Unknown	423 (62.5)	82 (59.9)	341 (63.3)	
Radiation therapy				0.1898
Irradiation	86 (12.7)	13 (9.5)	73 (13.5)	
No irradiation	590 (87.3)	124 (90.5)	466 (87.3)	
Steroid use at ICI treatment initiation				0.1628
Yes	53 (7.8)	7 (5.1)	46 (8.5)	
No	623 (92.2)	130 (94.9)	493 (91.5)	
ICI type				0.0418
Nivolumab	519 (76.7)	96 (70.1)	423 (78.5)	
Pembrolizumab	157 (23.2)	41 (29.9)	116 (21.5)	

*Note*: Categorical data are presented as numbers (percentages) and compared using the chi‐square test and Fisher's exact test. Continuous data are presented as medians (ranges) and compared using the Wilcoxon rank‐sum test. The *p*‐values were calculated by comparing subjects in the elderly and younger groups.

Abbreviations: ECOG, the Eastern Cooperative Oncology Group; ICI, immune‐checkpoint inhibitor; No., number; PD‐L1, programmed death‐ligand‐1.

^a^ *p* <0.05 was considered statistically significant.

### 3.2 Clinical outcomes

**FIGURE 1:**
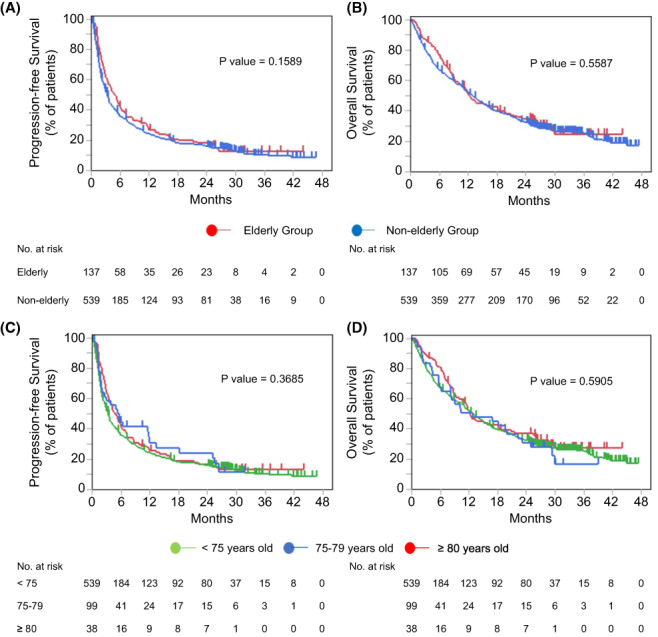
PFS and OS in the elderly (≥75 years) and younger (<75 years) groups. Kaplan–Meier curves of (A) PFS and (B) OS according to the patient's age. PFS and OS in the patients with ≥80 years old, 75‐79 years old, <75 years old groups. Kaplan‐Meier curves of (C) PFS and (D) OS according to the patient's age. OS, overall survival; PFS, progression‐free survival.

**FIGURE 2:**
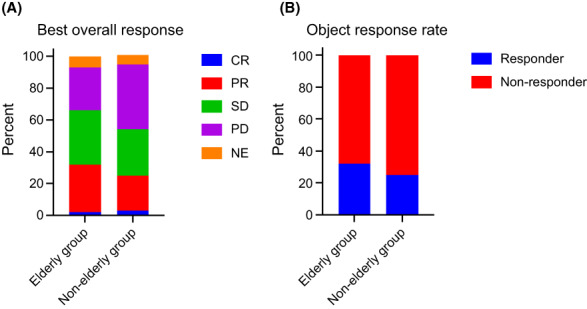
Treatment responses in the elderly and younger groups. (A) Best overall response of the elderly group was significantly better than that of the younger group (*p* = 0.0339). (B) Objective response rate was not significantly different between the two groups (*p* = 0.1028). CR, complete response; NE, not evaluable; PD, progressive disease; PR, partial response; SD, stable disease.

The median interval from the beginning of the initial ICI administration to the data cutoff was 34.3 months (range, 24.1–47.8). All surviving patients were followed up for at least 2 years from the initial ICI administration. The median PFS in the elderly group (4.8 months; 95% CI, 3.5–6.1) was similar to that in the younger group (3.3 months; 95% CI, 2.8–3.7; *p* = 0.1589; Figure 1A). The median OS in the elderly group (median, 12.3 months; 95% CI, 9.9–18.2) was also similar to that in the younger group (12.7 months; 95% CI, 11.6–14.3; *p* = 0.5587; Figure 1B). Regarding the tumor responses in the elderly group, the objective response rate (ORR) was 32.1%. Three (2.2%) patients achieved a CR, and 41 (29.9%) achieved a PR. A trend toward a higher ORR in the elderly group than in the younger group was observed (32.1% vs. 24.9%, *p* = 0.09) (Figure 2).

To further confirm that age was not a prognostic factor for the response to ICI therapy, we changed the cutoff age and divided the patients into three age groups: <75 years (*n* = 541), 75–79 years (*n* = 97), and ≥ 80 years (*n* = 38). The PFS rates of these three groups were 3.26, 4.59, and 5.27 months, respectively (*p* = 0.3685), and the OS rates were 13.0, 12.3, and 10.2 months, respectively (*p* = 0.5905), as shown in Figure 1C,D. The PFS and OS rates of the three groups were comparable and not significantly different.

**FIGURE 3:**
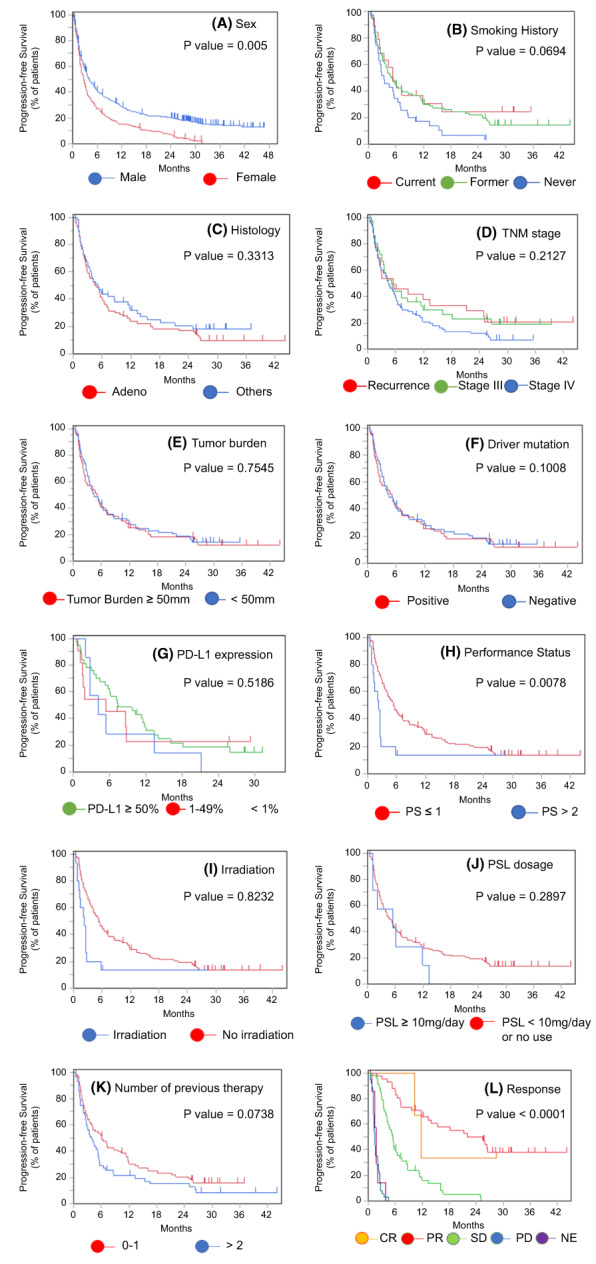
PFS in elderly patients according to subgroup analysis. Kaplan–Meier curves for PFS in elderly patients according to (A) sex, (B) smoking history, (C) histology, (D) TNM stage, (E) tumor burden, (F) driver mutation, (G) PD‐L1 expression, (H) performance status, (I) irradiation, (J) prednisolone dosage, (K) number of previous therapies, and (L) best response to ICI treatment. CR, complete response; NE, not evaluable; PD, progressive disease; PFS, progression‐free survival; PR, partial response; PS, performance status; PSL, prednisolone; SD, stable disease; TNM, tumor–node–metastasis.

**FIGURE 4:**
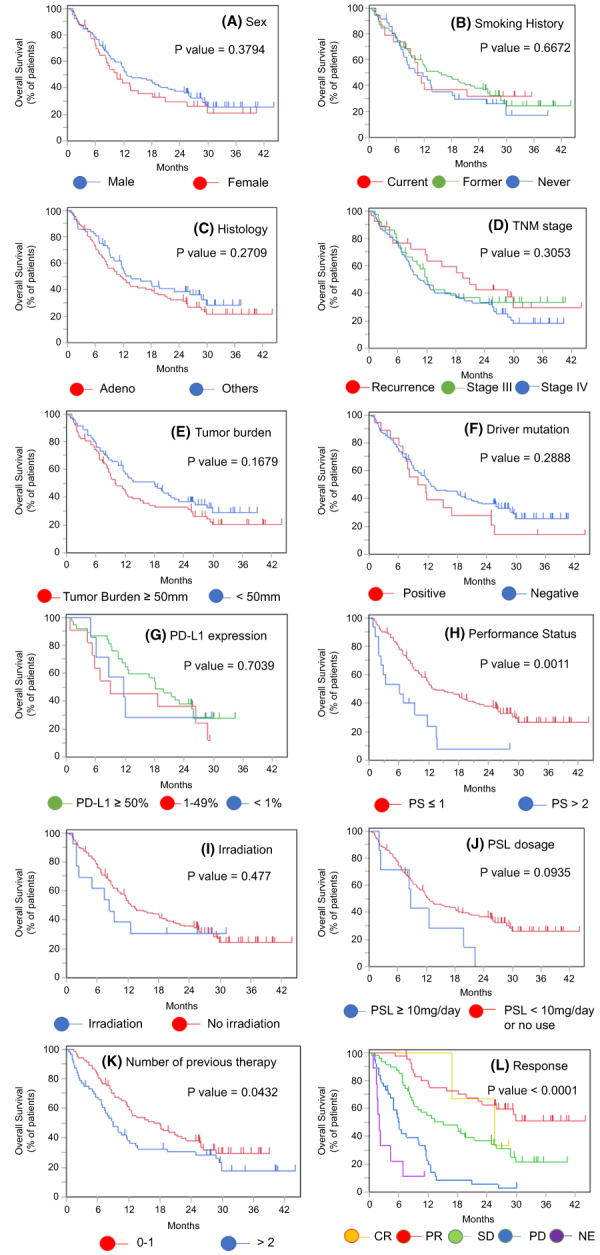
OS in elderly patients according to subgroup analysis. Kaplan–Meier curves for OS in elderly patients according to (A) sex, (B) smoking history, (C) histology, (D) TNM stage, (E) tumor burden, (F) driver mutation, (G) PD‐L1 expression, (H) performance status, (I) irradiation, (J) prednisolone dosage, (K) number of previous therapies, and (L) best response to ICI treatment. CR, complete response; NE, not evaluable; OS, overall survival; PD, progressive disease; PFS, progression‐free survival; PR, partial response; PS, performance status; PSL, prednisolone; SD, stable disease; TNM, tumor–node–metastasis.

Next, we investigated the prognostic factors in elderly patients. Univariate Cox proportional hazard regression analysis revealed that male sex, smoking, ECOG PS ≤1 at the time of ICI treatment initiation, tumor response, and treatment discontinuation due to irAEs were associated with a favorable PFS. We performed a multivariate Cox proportional hazards regression analysis with these five variables in terms of PFS. Sex (HR = 2.21; 95% CI, 1.31–3.75; *p* = 0.0041), ECOG PS (HR = 2.15; 95% CI, 1.19–3.89; *p* = 0.0209), and treatment discontinuation due to irAEs (HR = 0.56; 95% CI, 0.36–0.88, *p* = 0.0079) were significantly associated with a better PFS (Table 2). Univariate Cox proportional hazard regression analysis revealed that the treatment line in which ICI had been administered (1st or 2nd line), ECOG PS ≤1 at the time of ICI treatment initiation, a low NLR (<5), and treatment discontinuation due to irAEs were related to favorable OS (Table 3). We also performed a multivariate Cox proportional hazard regression analysis with the treatment lines in which ICI had been administered, the ECOG PS, the NLR, and treatment discontinuation due to irAEs to study their interactions with the OS. Significantly worse OS was associated with the treatment line in which ICI had been administered (1st or 2nd; HR = 1.72; 95% CI, 1.14–2.59; *p* = 0.011) and ECOG PS (HR = 2.06; 95% CI, 1.07–3.96; *p* = 0.0418), whereas significantly better OS was associated with the occurrence of irAEs leading to treatment discontinuation (HR = 0.56; 95% CI, 0.33–0.93; *p* = 0.02). Patients with a low NLR tended to have a better OS, but the difference was not statistically significant (HR = 1.40; 95% CI, 0.81–2.40; *p* = 0.2375). The Kaplan–Meier curves for PFS and OS are presented according to the subgroup in Figures 3 and 4.

**TABLE 2:** Univariate and multivariate analyses of PFS in the elderly group.

			Univariate			Multivariate		
Parameter	Category	No. of patients	HR	95% CI	*p*‐value^a^	HR	95% CI	*p*‐value^a^
Sex	Female (vs. male)	40 (97)	1.97	1.33–2.93	0.0007	2.21	1.31–3.75	0.0041
Smoking status	Smoker (vs. never)	102 (35)	0.63	0.42–0.95	0.0266	0.92	0.53–1.60	0.7802
Pack‐years	≥30 (vs. <30)	69 (68)	0.77	0.53–1.11	0.1612			
Histology	Ad (vs. others)	81 (56)	1.21	0.83–1.76	0.3333			
Stage at diagnosis	Stage IV (vs. others)	75 (62)	1.4	0.96–2.03	0.0812			
Tumor burden	<50 mm (vs. ≥50 mm)	67 (70)	0.94	0.65–1.36	0.7552			
Treatment line	≥3rd (vs. 1st or 2nd)	56 (81)	1.4	0.97–2.03	0.076			
ECOG Performance status	≥2 (vs. 0 or 1)	15 (122)	2.17	1.21–3.89	0.0096	2.15	1.19–3.89	0.0209
PD‐L1 status	1–49% (vs. <1%)	11 (7)	0.73	0.27–2.03	0.5525			
	≥50% (vs. <1%)	37 (18)	0.64	0.28–1.47	0.2962			
Response category	CR (vs. others)	3 (134)	0.15	0.09–0.25	<0.0001	0.52	0.13–2.12	0.3083
NLR	≥5 (vs. <5)	28 (109)	1.2	0.74–1.95	0.4662			
Radiation therapy	With (vs. without)	13 (124)	1.08	0.56–2.06	0.8237			
Treatment discontinuation due to AE	With (vs. without)	34 (103)	0.55	0.35–0.85	0.0075	0.56	0.36–0.88	0.0079

*Note*: Hazard ratios (HRs) and 95% confidence intervals (CIs) were estimated using the Cox proportional hazards regression model. Without considering the results of the univariate analysis, the factors considered important based on previous reports and a medical standpoint were included in the multivariate analysis.

Abbreviations: AE, adverse event; CR, complete response; ECOG, the Eastern Cooperative Oncology Group; NLR, neutrophil‐lymphocyte ratio; No, number; PD‐L1, programmed death‐ligand‐1; PFS, progression‐free survival.

^a^ *p* <0.05 was considered statistically significant.

**TABLE 3:** Univariate and multivariate analyses of OS in the elderly group.

			Univariate			Multivariate		
Parameter	Category	No. of patients	HR	95% CI	*p*‐value^a^	HR	95% CI	*p*‐value^a^
Sex	Female (vs. male)	40 (97)	1.21	0.78–1.88	0.3803			
Smoking status	Smoker (vs. never)	102 (35)	0.81	0.52–1.28	0.3773			
Pack‐years	≥30 (vs. <30)	69 (68)	1.02	0.68–1.52	0.9204			
Histology	Ad (vs. others)	81 (56)	1.26	0.83–1.90	0.2678			
Stage at diagnosis	Stage IV (vs. others)	75 (62)	1.36	0.90–2.03	0.26			
Tumor burden	<50 mm (vs. ≥50 mm)	67 (70)	0.94	0.65–1.36	0.1691			
Treatment line	≥3rd (vs. 1st or 2nd)	56 (81)	1.51	1.01–2.26	0.0471	1.72	1.14–2.59	0.011
ECOG Performance status	≥2 (vs. 0 or 1)	15 (122)	2.6	1.43–4.70	0.0016	2.06	1.07–3.96	0.0418
PD‐L1 status	1–49% (vs. <1%)	11 (7)	1.18	0.39–3.52	0.7702			
	≥50% (vs. <1%)	37 (18)	0.79	0.30–2.07	0.6348			
Response category	CR (vs. others)	3 (134)	0.63	0.39–6.41	0.4922			
NLR	≥5 (vs. <5)	28 (109)	1.81	1.12–2.91	0.0218	1.4	0.81–2.40	0.2375
Radiation therapy	With (vs. without)	13 (124)	1.28	0.65–2.55	0.4929			
Treatment discontinuation due to AE	With (vs. without)	34 (103)	0.56	0.34–0.91	0.014	0.56	0.33–0.93	0.02

*Note*: Hazard ratios (HRs) and 95% confidence intervals (CIs) were estimated using the Cox proportional hazards regression model. Without considering the results of the univariate analysis, the factors considered important based on previous reports and a medical standpoint were included in the multivariate analysis.

Abbreviations: AE, adverse event; CR, complete response; ECOG, the Eastern Cooperative Oncology Group; NLR, neutrophil‐lymphocyte ratio; No, number; OS, overall survival; PD, progressive disease; PD‐L1, programmed death‐ligand‐1.

^a^ Results were considered statistically significant at *p* < 0.05.

### 3.3 Safety

No significant difference was found in the proportion of patients who discontinued ICI treatment because of irAEs between the elderly and younger groups (24.8% vs. 19.3%, *p* = 0.1557). In addition, there was no significant difference in the incidence of grade‐5 irAEs between these two groups (0% vs. 0.9%, *p* = 0.5889). Pneumonitis was reported in 16 patients (11.7%) in the elderly group and 53 patients (8.2%) in the younger group (Table 4), making it the most frequently reported irAEs. The profile and severity of irAEs are provided in Table 5. There were some cases in which ICI was discontinued due to irAEs in grades 1–2. Most of them were due to pneumonitis, suggesting that in clinical practice, ICI may have been withdrawn relatively early due to the risk of serious exacerbation, especially in elderly patients.

**TABLE 4:** Frequencies and types of irAEs in elderly and younger patients.

	No. (%)		
	Elderly group	Younger group	*p*‐value^a^
Lead to treatment discontinuation	34/137 (24.8)	104/539 (19.3)	0.1557
Death due to irAEs	0 (0)	5 (0.9)	0.5889
Profile of irAEs			0.9187
Pneumonitis	16 (11.7)	53 (8.2)	
Endocrine disfunction	6 (4.4)	14 (2.1)	
Neural and muscular disfunction	4 (2.9)	12 (1.8)	
Gastrointestinal disfunction	4 (2.9)	11 (1.7)	
Skin disfunction	1 (0.7)	7 (1.1)	
Blood toxicity	1 (0.7)	1 (0.2)	
Others	2 (1.4)	6 (0.9)	

Abbreviation: irAEs, Immune‐related adverse events.

^a^ *p* < 0.05 was considered statistically significant.

**TABLE 5:** Profile and severity of irAEs in patients who discontinued treatment due to irAEs.

Grade of irAEs	No. of patients	Profile of irAEs (number of patients)
Grade 1	4	Pneumonitis (2), Hypothyroidism (1), Pituitarytis (1)
Grade 2	9	Pneumonitis (6), Hypothyroidism (1), Peripheral neuropathy (1), Fatigue (1)
Grade 3	10	Pneumonitis (7), Musculoskeletal disorder (2), Adrenal insufficiency (1)
Grade 4	5	Gastrointestinal bleeding (1), Colitis (1), Hepatitis (1) Platelet reduction (1), Type 1 Diabetes mellitus (1)
Grade 5	0	
Unknown	6	Pneumonitis (1), Hepatitis (1), Hypothyroidism (1), Encephalitis (1), Infusion reaction (1), Pemphigoid (1)

Abbreviation: irAE, immune‐related adverse event.

### 3.4 Relationship between irAEs and the survival benefit

**FIGURE 5:**
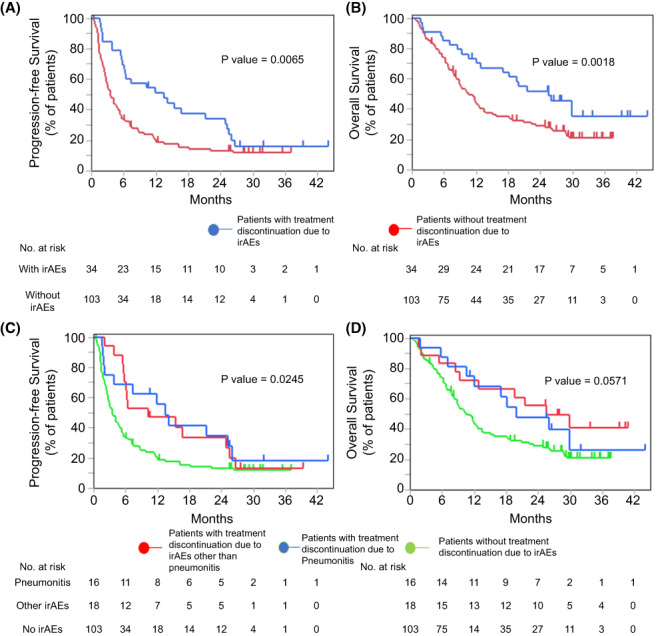
PFS and OS in the elderly groups in relation to irAEs. Kaplan–Meier curves for (A) PFS and (B) OS according to the reason for treatment discontinuation (disease progression or irAEs). (C) PFS and (D) OS according to whether patients discontinued treatment due to irAEs. irAEs, immune‐related adverse events; OS, overall survival; PFS, progression‐free survival.

Multivariate Cox proportional hazard regression analysis showed that patients who discontinued treatment due to irAEs had a good prognosis; therefore, we analyzed the relationship between irAEs and survival outcomes. First, we compared patients who discontinued ICI therapy due to irAEs with those who did not discontinue treatment due to irAEs. The median PFS in the irAE group (median, 14.1 months vs. 7.49 months; *p* = 0.0065) and OS in the irAE group (median, 20.5 months vs. 14.3 months; *p* = 0.0180) (Figure 5A,B) were significantly better than in patients without irAEs.

**FIGURE 6:**
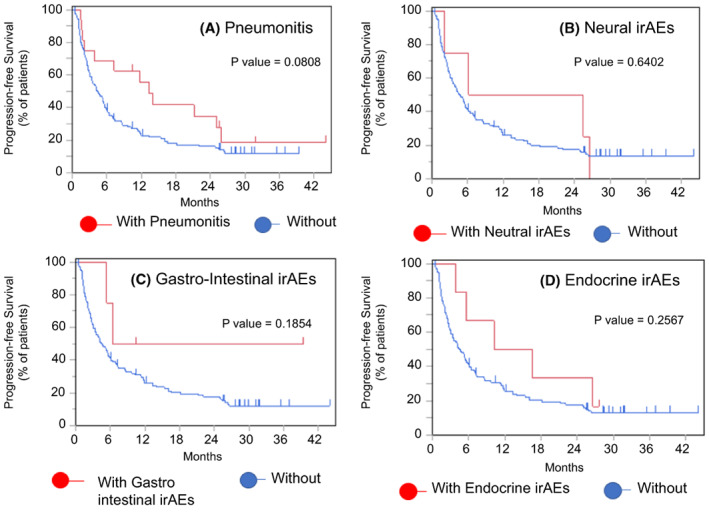
PFS in patients with/without treatment discontinuation due to irAEs. Kaplan–Meier curves of PFS in elderly patients according whether treatment was discontinued due to (A) pneumonitis, (B) neural irAEs, (C) gastrointestinal irAEs, and (D) endocrine irAEs. irAE, immune‐related adverse event.

**FIGURE 7:**
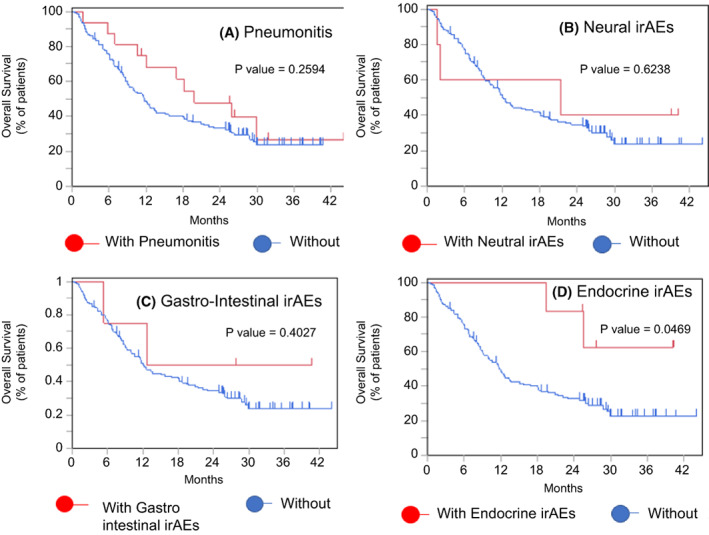
OS in patients with/without treatment discontinuation due to irAEs. Kaplan–Meier curves for OS in elderly patients according to whether treatment was discontinued due to (A) pneumonitis, (B) neural irAEs, (C) gastrointestinal irAEs, and (D) endocrine irAEs. irAE, immune‐related adverse event.

Among the different types of irAEs, pneumonitis was one of the fatal adverse events noted during ICI therapy, and this condition may lead to a worse prognosis because of limited treatment options. However, in this study, even patients who discontinued ICI treatment due to pneumonitis had better PFS (median, 14.3 months vs. 8.43 months; *p* = 0.0808) and OS (median, 19.8 months vs. 11.7 months; *p* = 0.2594) rates than those who did not discontinue treatment due to irAEs (Figure 5C,D). The number of patients with irAEs other than pneumonitis (i.e., those with endocrine, gastrointestinal, neural, or muscular disorders) was very low in each group, but generally, they also had a better prognosis than patients who did not discontinue treatment due to irAEs (Figures 6 and 7).

### 3.5 NLR as a marker of good prognosis and patient selection in elderly patients

**FIGURE 8:**
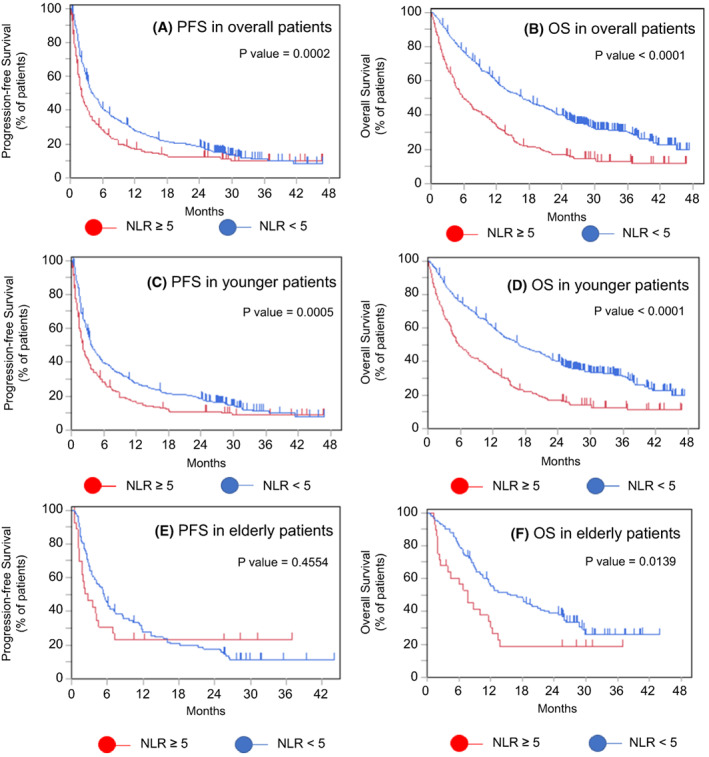
Relationships between PFS and OS and the NLR. Kaplan–Meier curves for (A) PFS and (B) OS in all patients, (C) PFS and (D) OS in elderly patients, and (E) PFS and (F) OS in younger patients. NLR, neutrophil‐lymphocyte ratio; OS, overall survival; PFS, progression‐free survival.

We investigated the possibility of selecting elderly patients based on routine blood tests that are used in our daily medical practice. Among the findings from the blood tests, the NLR has been suggested to correlate with prognosis in many cancer types, although the cutoff value remains controversial.^25^, ^26^, ^27^, ^28^, ^29^, ^30^ Even when restricted to NSCLC, there is controversy regarding the cutoff of NLR. In a meta‐analysis by Wang et al., the NLR ranged from 2.11 to 5.90 with regard to the relationship between PFS and prognosis, and from 2.11 to 6.50 with regard to the relationship between OS and NLR.^30^ In the present study, we set the NLR cutoff at 5, in line with a previous study that investigated the relationship between ICI alone and prognosis in lung cancer with good results.^26^, ^27^ Significantly more elderly patients had an NLR of <5 (Table 6A). We examined the overall population (Figure 8A,B), including younger patients (Figure 8C,D), using a cutoff value of 5 and found significant differences in the PFS and OS rates (overall PFS: median, 3.6 months vs. 1.8 months, *p* = 0.0002; OS: median, 16.9 months vs. 6.0 months, *p* < 0.0001; PFS in younger patients: median, 3.7 months vs. 2.1 months, *p* = 0.0005; OS in younger patients: median, 16.9 months vs. 5.7 months, *p* < 0.0001). Our univariate and multivariate Cox proportional hazard regression analyses revealed that a low NLR was still significantly associated with a good prognosis (data not shown). In younger patients, only a low NLR was significantly associated with a better OS (data not shown). In elderly patients, although PFS did not differ significantly (5.4; 95% CI, 3.8–7.0 months vs. 2.7; 95% CI, 1.3–4.5 months; *p* = 0.4554) (Figure 8E), the OS was significantly better in the population with an NLR of <5 (15.7; 95% CI, 11.3–22.2 months vs. 7.6; 95% CI, 2.5–12.0 months; *p* = 0.0139) (Figure 8F). However, this variable was not significant after performing multivariate analysis. In addition, the results of our study revealed that significantly more patients with an ECOG PS of 0–1 had an NLR of <5 than did those with an ECOG PS of ≥2 (82.7% vs. 53.3%, *p* = 0.0144) (Table 6B). Significant correlations were also found between the NLR and the relative level of disease control (CR, PR, and SD) (73.4% vs. 39.3%, *p* = 0.019; Table 6C). These data suggest that the NLR is an indicator of a good immune status that facilitates long‐term responses to ICI therapy in the elderly. Thus, the NLR of elderly patients may be a potential marker for selecting patients that are likely to respond to ICI treatment.

**TABLE 6:** Relationships between patient characteristics and the NLR.

	No. (%)		
	Elderly group	Younger group	*p*‐value^a^
(A) Difference in NLR between elderly and younger patients			
NLR ≧5	29 (21.2)	173 (32.1)	0.0159
NLR <5	108 (78.8)	366 (67.9)	

	No. (%)		
	NLR ≥5	NLR <5	*p*‐value^a^
(B) Relationship between NLR and PS in elderly patients			
PS 0–1	21 (15.3)	101 (73.7)	0.0144
PS ≥2	7 (5.1)	8 (5.9)	
(C) Relationship between NLR and best response in elderly patients			
CR	0 (0)	3 (2.8)	0.0019
PR	8 (28.6)	33 (30.3)	
SD	3 (10.7)	44 (40.3)	
PD	12 (42.9)	25 (22.9)	
NE	5 (17.8)	4 (3.7)	

Abbreviations: CR, complete response; NE, not evaluable; NLR, Neutrophil‐Lymphocyte ratio; PD, progressive disease; PR, partial response; PS, performance status; SD, stable disease.

^a^ *p* < 0.05 was considered statistically significant.

## 4 DISCUSSION

In our study, ICI is as effective in elderly patients as in younger patients. Because both elderly and younger patients were enrolled in this study, we were able to directly compare the efficacy and safety of ICI in elderly patients with those in younger patients. Only a few studies have been conducted with real‐world data used to directly compare therapeutic responses in elderly and younger patients.^16^, ^17^ In a clinical trial setting, Nosaki et al. found that the safety and efficacy of pembrolizumab monotherapy in patients ≥75 years were comparable to those in the general population, based on a pooled analysis of the data from the Keynote‐010, Keynote‐024, and Keynote‐042 studies.^31^ However, relatively few elderly patients were enrolled in their pivotal study, and most of them might have had a better ECOG PS with fewer comorbidities than patients in the real world. In this study, we compared the efficacy of ICI monotherapy in elderly people with that in non‐elderly patients in a real‐world setting. Although this difference was not statistically significant, a better ORR was found in the elderly group. Therefore, ICI therapy in elderly patients was as effective as that in younger patients.

Next, we examined the efficacy and safety of ICI therapy in patients aged ≥80 years. In today's aging society, an increasing number of elderly patients aged ≥80 years are becoming possible candidates for anticancer therapy. Generally, the profile of this age group differs from that of patients aged ≥75 years in terms of number of comorbidities, altered drug disposition, and polypharmacy. However, efficacy and safety studies on this population are limited. Nebhan et al.^18^ reported that median PFS was 6.7 months and OS was 10.9 months in 345 patients aged ≥80 years. In our study, we enrolled 38 patients ≥80 years of age, and PFS and OS in these patients were comparable to those of the patients aged <80 years old. The study of Nebhan et al. differs from ours in that it used a cutoff age of 85 years, but both studies suggest that even very elderly patients with good ECOG PS respond to ICI therapy as well as younger patients. Contrary to these findings, Lichtenstein et al. reported that patients aged ≥80 years had a worse median PFS than patients aged 60–69 or 70–79 years (1.64 vs. 2.53 vs. 3.75 months; *p* = 0.055) and a significantly worse median OS than patients aged 60–69 years or 70–79 years (3.62 vs. 14.56 vs. 12.92 months; *p* = 0.011).^17^ This discrepancy may be due to the small number of patients and the poor ECOG PS in Lichtenstein's study, where only 28 patients ≥80 years were enrolled, 42.8% of whom had an ECOG PS of ≥2.

We then explored prognostic factors in the elderly patients. Univariate and multivariate analyses showed that patients who discontinued treatment because of irAE had an even better prognosis than those who were able to continue treatment without serious adverse events. Some past studies have investigated the relationship between the occurrence of irAEs and better prognosis.^32^, ^33^, ^34^, ^35^, ^36^, ^37^, ^38^ When the prognostic impact of each type of irAE was examined, many of the previous reports focused on the relationship with skin disorders.^37^, ^38^ For example, Kimberly et al. retrospectively examined 7008 patients who developed skin irAEs after ICI therapy in many types of cancers, including NSCLC, and found that many types of skin irAEs (i.e., pruritus, drug eruption, xerosis, and nonspecific rashes) were associated with a lower mortality rate.^38^ In contrast, although the number of reports on pneumonitis is not large, there have been reports in recent years showing a relationship between the occurrence of pneumonitis and good prognosis.^35^, ^36^ For example, Ono et al. reported that patients who had ICI‐derived pneumonitis had significantly longer PFS (18.9 months vs. 3.9 months, *p* < 0.01) and OS (27.4 months vs. 14.8 months, *p* = 0.003). In addition, 25% of patients with ICI‐induced pneumonitis survived for more than 300 days after treatment discontinuation.^36^ However, these pneumonitis studies were analyses of populations that included young people, and studies restricted to the elderly are extremely scarce. Our study shows that the occurrence of irAEs is correlated with good prognosis in elderly patients and in younger patients. We limited our study to adverse events occurring in more than 10% of elderly patients and examined the prognostic relationship (Figures 6 and 7). Although the small number of cases precluded any definitive conclusions, the prognosis tended to be better for all types of irAE. Even patients who discontinued treatment due to pneumonitis had a similar prognosis compared to patients with other irAEs. Regarding the prognostic impact of irAE treatment, Yamaguchi et al. showed that the use of steroids for irAEs was associated with a longer PFS in elderly patients.^15^ This finding suggests that appropriate treatment for irAEs may lead to a better prognosis. When looking at the association between irAEs and prognosis, the influence of immortal time bias cannot be ruled out.^39^ However, given the large difference in the survival rate at 2 years (50% vs. 24.2%), our study shows at least non‐negative prognostic effects in elderly patients (Figure 5B), whose irAEs are of greater concern, supporting the aggressive administration of ICIs in elderly patients.

NLR was found to have clinical utility as a prognostic factor for ICI therapy in the elderly, with OS significantly longer in elderly patients with NLR <5. There was no significant difference in PFS, but this may be due to low power due to the small number of patients in our cohort. Therefore, it will be desirable to confirm the validity of the NLR in a larger population in the future. Moreover, we not only identified NLR as a prognostic marker but also found the relationship between ECOG PS, disease control rate, and NLR in elderly patients. Since elderly patients comprise a very diverse group, their immune status cannot be evaluated from clinical information easily. As a result, determining NLR may be an easy approach for accessing the immune status for ICI therapy.

This study has several limitations. First, because of its retrospective nature, we could not collect clinical information regarding comorbidities and patients' daily activities. Therefore, we could not evaluate certain distinct characteristics in elderly people. We cannot rule out the possibility that these factors, presently not considered, may influence the prognosis of ICI treatment in elderly patients. Second, some data, such as the PD‐L1‐expression status, were insufficient. Third, the elderly and younger patient groups were not well balanced; that is, the elderly group included more patients with a better ECOG PS.

In conclusion, ICI therapy was as effective in elderly NSCLC patients as it was in non‐elderly patients; therefore, age is not a reason to refrain from administering ICIs. Importantly, treatment discontinuation due to irAEs may be a good prognostic marker for elderly patients, and efforts should be focused on the proper management of irAEs to achieve long‐term survival.

## AUTHOR CONTRIBUTIONS

**Daisuke Morinaga:** Conceptualization (equal); data curation (equal); formal analysis (lead); investigation (lead); project administration (equal); visualization (lead); writing – original draft (lead). **Hajime Asahina:** Conceptualization (equal); data curation (equal); investigation (equal); methodology (equal); project administration (equal); supervision (lead); writing – review and editing (lead). **Shotaro Ito:** Conceptualization (equal); data curation (equal); formal analysis (equal); investigation (equal); project administration (equal); writing – review and editing (equal). **Osamu Honjo:** Investigation (equal); writing – review and editing (equal). **Hisashi Tanaka:** Investigation (equal); writing – review and editing (equal). **Ryoichi Honda:** Investigation (equal); writing – review and editing (equal). **H. Yokouchi:** Investigation (equal); writing – review and editing (equal). **Keiichi Nakamura:** Investigation (equal); writing – review and editing (equal). **Kei Takamura:** Investigation (equal); writing – review and editing (equal). **Fumihiro Hommura:** Investigation (equal); writing – review and editing (equal). **Yasutaka Kawai:** Investigation (equal); writing – review and editing (equal). **Kenichiro Ito:** Investigation (equal); writing – review and editing (equal). **Noriaki Sukoh:** Investigation (equal); writing – review and editing (equal). **Keiki Yokoo:** Investigation (equal); writing – review and editing (equal). **Ryo Morita:** Investigation (equal); writing – review and editing (equal). **Toshiyuki Harada:** Investigation (equal); writing – review and editing (equal). **Taichi Takashina:** Investigation (equal); writing – review and editing (equal). **Tomohiro Goda:** Investigation (equal); writing – review and editing (equal). **Hirotoshi Dosaka‐Akita:** Investigation (equal); writing – review and editing (equal). **Hiroshi Isobe:** Investigation (equal); writing – review and editing (equal).

## FUNDING INFORMATION

This research did not receive any specific grant from funding agencies in the public, commercial, or not‐for‐profit sectors.

## CONFLICT OF INTEREST STATEMENT

Dr. Hajime Asahina reported receiving lecture fees from Chugai Pharmaceutical. Dr. Osamu Honjo reported lecture fees from Bristol‐Myers Squib K.K. Dr. Hisashi Tanaka reported receiving lecture fees from Chugai Pharmaceutical and Ono Pharmaceutical Co., Ltd during the conduct of the study. Dr. Hiroshi Yokouchi reported receiving lecture fees from AstraZeneca, and grants from Taiho Pharmaceutical, Sanofi, Bristol‐Myers Squibb, MSD, Takeda Pharmaceutical, Daiichi‐Sankyo, and Chugai Pharmaceutical during the conduct of the study. The other authors declare no conflicts of interest.

## ETHICS APPROVAL STATEMENT

This study was carried out in accordance with The Code of Ethics of the World Medical Association (Declaration of Helsinki). This study was approved by the Hokkaido University Hospital Ethics Committee (approval no. 022–0123).

## PATIENT CONSENT STATEMENT

The need for informed consent was waived because anonymized data were analyzed.

## CLINICAL TRIAL REGISTRATION

This study was registered at UMIN‐CTR (UMIN000041403).

## Data Availability

The data that support the findings of this study are available on request from the corresponding author. The data are not publicly available due to privacy or ethical restrictions
